# Crystallization of TiO_2_ Nanotubes by *In Situ* Heating TEM

**DOI:** 10.3390/nano8010040

**Published:** 2018-01-14

**Authors:** Alberto Casu, Andrea Lamberti, Stefano Stassi, Andrea Falqui

**Affiliations:** 1 King Abdullah University of Science and Technology (KAUST), Biological and Environmental Sciences and Engineering (BESE) Division, NABLA Lab, 23955-6900 Thuwal, Saudi Arabia; 2 Department of Applied Science and Technology, Politecnico di Torino, Corso Duca degli Abruzzi 24, 10129 Torino, Italy; andrea.lamberti@polito.it (A.L.); stefano.stassi@polito.it (S.S.)

**Keywords:** TiO_2_ amorphous nanotubes, high resolution transmission electron microscopy, *in situ* transmission electron microscopy, amorphous-crystalline phase transition, electron beam effects, anodic oxidation

## Abstract

The thermally-induced crystallization of anodically grown TiO_2_ amorphous nanotubes has been studied so far under ambient pressure conditions by techniques such as differential scanning calorimetry and *in situ* X-ray diffraction, then looking at the overall response of several thousands of nanotubes in a carpet arrangement. Here we report a study of this phenomenon based on an *in situ* transmission electron microscopy approach that uses a twofold strategy. First, a group of some tens of TiO_2_ amorphous nanotubes was heated looking at their electron diffraction pattern change versus temperature, in order to determine both the initial temperature of crystallization and the corresponding crystalline phases. Second, the experiment was repeated on groups of few nanotubes, imaging their structural evolution in the direct space by spherical aberration-corrected high resolution transmission electron microscopy. These studies showed that, differently from what happens under ambient pressure conditions, under the microscope’s high vacuum (*p* < 10^−5^ Pa) the crystallization of TiO_2_ amorphous nanotubes starts from local small seeds of rutile and brookite, which then grow up with the increasing temperature. Besides, the crystallization started at different temperatures, namely 450 and 380 °C, when the *in situ* heating was performed irradiating the sample with electron beam energy of 120 or 300 keV, respectively. This difference is due to atomic knock-on effects induced by the electron beam with diverse energy.

## 1. Introduction

Titanium dioxide (TiO_2_), even in form of nanotubes, is a well-known material due to its physical and chemical properties and related applications in many diverse fields, such as non-linear optics, photocatalysis, energy storage, optoelectronics, transport-related phenomena, dye-synthesized solar cells, mesoporous structures and film formation [1,2,3,4,5,6,7,8,9,10,11,12,13,14]. TiO_2_ can be found as an amorphous and in several, different crystalline polymorphs, being the most known anatase (tetragonal), rutile (tetragonal) and brookite (orthorhombic). Besides, TiO_2_ amorphous nanotubes (TANs) prepared by anodic oxidation of a titanium surface also deserved large interest due to their further and diverse applications, again such as energy harvesting and storage, optoelectronics, photocatalysis, water splitting, and as an effective biomaterial [15,16,17,18]. Indeed, as-grown TANs can be easily crystallized in the desired phase by simple thermal treatment.

So far, few detailed studies of both amorphous TiO_2_ films of different thickness and TAN crystallization have been published [19,20,21,22,23]. Most of them are based on techniques, such as X-ray diffraction (XRD) and differential scanning calorimetry (DSC), that show the overall behavior of a multitude of objects subject to a thermal ramp performed under standard pressure conditions (10^5^ Pa). More in detail, the studies performed on amorphous TiO_2_ films deposited by magnetron sputtering onto monocrystalline silicon substrate showed that both the initial crystallization temperature and the time needed to crystallize the whole film depend strongly on its features: the higher its thickness, the lower both the initial crystallization temperature and the time needed for the film’s complete crystallization [19,20]. Among these works, the minimum transition temperature found for the crystallization of a thick TiO_2_ film (800 nm-thick) was reported as being about 180 °C [10]. Moreover, once the amorphous films started to crystallize onto the silicon substrate, the zones close to it evolved to the rutile phase, and those far from it to anatase. Besides, after their formation the crystalline domains kept a small size (i.e., less than 10 nm) up to a quite high temperature; also, a substantial increase in temperature was needed to promote the transition from anatase to rutile for the portion of the films not in proximity of the substrate [21]. A quite similar XRD- and DSC-based approach was followed to investigate the crystallization of highly ordered TANs grown on a titanium substrate [22]. In the latter case, it was shown that at 350 °C TANs started to crystallize as anatase, with a mean crystalline domain size around 20 nm, while rutile appeared only for temperatures higher than 600 °C. Finally, an important work was published showing the crystallization of TANs studied by both thermal gravimetric analysis/differential thermal analysis, XRD and *ex situ* transmission electron microscopy (TEM) [23]. In this work, which concerns template-directed low temperature atomic layer deposition on an alumina template, physically confined TANs were prepared and thermally crystallized. In this case, it was clearly observed that TANs with a wall thickness of 5 nm, when confined, become crystalline at 400 °C taking the anatase structure, which is retained even if the nanotubes are heated up to 1000 °C. Besides, if the TANs are annealed with no further physical confinement thanks to the low-temperature programmed dissolution of the polymer template, they still keep their tubular shape (this phenomenon, due to their intrinsic curvature, being called “self-confinement”) and again take the anatase crystal phase at the same temperature of the confined ones, i.e., 400 °C, with some minority traces of the rutile phase being found at 1000 °C. 

Thus, even though the thermally-induced crystallization of TANs has been understood in terms of transition temperatures and final crystalline phases obtained, as in the case of the amorphous TiO_2_ films, these investigations did not unveil the effect of a very low external pressure on this phenomenon, nor the mechanism that leads to the crystal formation at the local scale under the abovementioned high vacuum conditions. 

Furthermore, even though some works were published concerning *in situ* studies of materials with some similarity to TANs for shape or composition [24,25,26,27], to our best knowledge the TANs thermally-induced crystallization was never investigated by an *in situ* TEM-based approach. Herein, the study of TAN crystallization has been then performed using this approach and following a twofold experimental strategy that allowed us to observe how this phenomenon occurs and evolves at a very local scale. The first part of this strategy consisted in heating *in situ* a group of several tens of TANs, while looking at their electron diffraction pattern and at its variation over time. This allowed the determination of both the starting temperature of crystallization and the evolution of the appearing crystalline phases. Second, the experiment was repeated on several groups of few nanotubes, imaging their structural change in the direct space by spherical aberration (C_s_)-corrected high resolution transmission electron microscopy (HRTEM). These studies permitted the determination that, differently from what happens under standard pressure, under the microscope’s vacuum (*p* < 10^−5^ Pa) the crystallization of TiO_2_ amorphous nanotubes starts from local small seeds of rutile and brookite, which then grow up with the increasing temperature. Besides, different starting temperatures of crystallization, namely 450 °C and 380 °C, were observed when the *in situ* heating was performed with electron beam energy of 100 and 300 keV, respectively. This difference is due to atomic knock-on effects induced by the electron beam with diverse energy. 

## 2. Results

**Figure 1:**
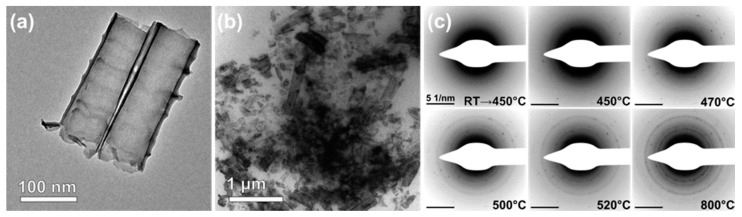
*In situ* selected area electron diffraction (SAED) heating experiment: (**a**) Representative (TiO2 amorphous nanotubes) TANs at room temperature RT; (**b**) The several micron-sized aggregate of TiO_2_ TANs chosen for the *in situ* heating; (**c**) SAED patterns of the aggregate reported in panel (**b**), recorded at different temperatures during the *in situ* heating experiment.

After deposition on the micro electro-mechanical system (MEMS) constituting the *in situ* holder grid, it was possible to determine that TANs displayed a mean diameter of 80 nm and mean length of 250 nm, as shown in Figure 1a,b, with a minority group of longer TANs (see Figure 1b). As a first experiment, the TANs’ structural evolution was studied by recording the two-dimensional electron diffraction (ED) patterns of a several micron-sized aggregate of TiO_2_ nanotubes, as displayed in Figure 1b, while increasing the temperature from room temperature (RT = 20 °C) to 800 °C. No structural evolution was observed in the amorphous TiO_2_ sample up to 450 °C, as evidenced by the lack of diffraction spots in the selected area electron diffraction (SAED) pattern. Between 450 and 520 °C a full structural evolution was observed and is shown in Figure 1c: the first low-intensity diffraction spots appeared at 450 °C, with a subsequent evolution of the patterns towards a full SAED pattern showing diffraction rings and spots compatible with the rutile phase by 470 °C. A subsequent increase in temperature to 500 °C resulted in the appearance of additional diffraction spots and rings, which were attributed to the presence of both the rutile and brookite phases, until a stable diffraction pattern was reached at 520 °C. Additional heating treatments up to 800 °C did not result in any further modification of the SAED patterns, which were also maintained after decreasing the temperature to RT.

**Figure 2:**
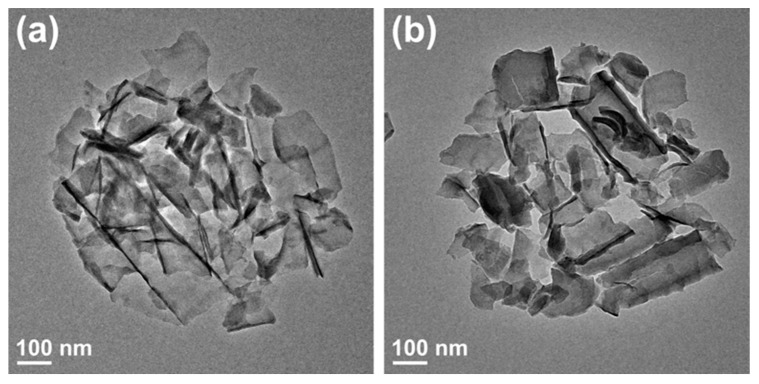
Low magnification transmission electron microscopy (TEM) images of micron-sized TANs: (**a**,**b**) Small aggregates of TANs recorded at RT and used for the *in situ* high resolution transmission electron microscopy (HRTEM) heating experiment.

*In situ* HRTEM analysis was then performed on a 300 kV Cs-corrected microscope, taking into account the structural information obtained by the previous experiment and studying the structural evolution of different aggregates against temperature at a very local scale. In fact, given the amorphous nature of TANs at room temperature, it was not possible to know *a priori* which ones would have been properly oriented for HRTEM imaging. Then, several aggregates constituted by a few TANs each were imaged and their positions were recorded before starting any heating ramp. Some of these aggregates are reported in Figure 2. Besides, the thermal treatment during the *in situ* HRTEM experiment was performed according to a slightly different procedure than that followed for the first ED-based experiment reported above. In fact, here the sample heating was performed up to a given temperature, then lowered to 100 °C during the acquisition of the corresponding HRTEM images and heated up again to a higher fixed temperature. This “sawtooth” thermal approach allowed the analysis of the structural evolution of the different zones under the same thermal conditions: the whole sample was subject to the temperature ramp, but the time needed to analyze the appropriately chosen zones was spent at low temperature, where no temperature-driven structural variation is expected, thus minimizing the occurrence of any further structural evolution in-between different zones. 

**Figure 3:**
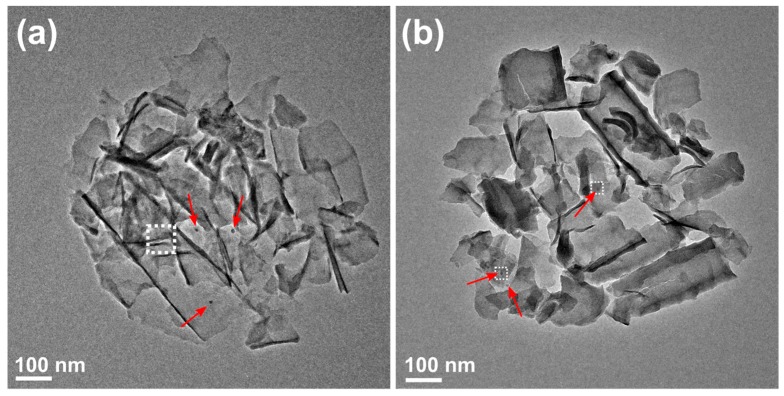

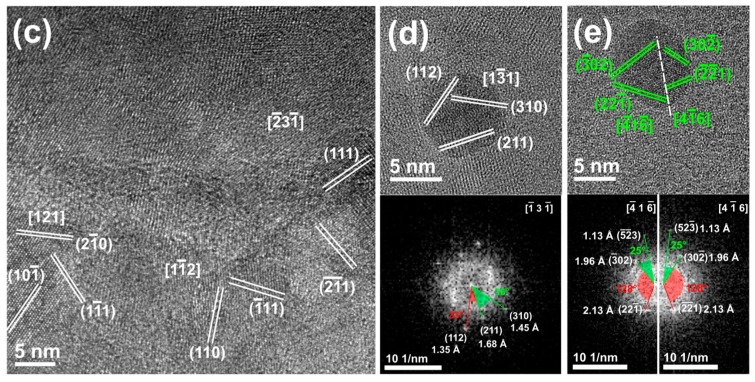
*In situ* HRTEM heating experiment of micron-sized TANs: (**a**,**b**) Evolution of the small aggregates of TANs reported in Figure 2, now recorded at 380 °C. The red arrows reported in panel **a** and **b** indicate the localization of several small crystalline seeds not observed in the panel (**a**,**b**) of Figure 2. The white dotted rectangles indicate the regions of interest analyzed by HRTEM and reported in panels **c**, **d** and **e**; (**c**) HRTEM image of a region with patched crystallization of rutile (in white) in the first aggregate (see the rectangle in panel 3**a**); (**d**) HRTEM image of a rutile seed (in white) in the second aggregate (see the rectangle on the left in panel and corresponding two-dimensional fast Fourier transform (2D-FFT) showing the expected interplanar distances and angular relationships, 3**b**); (**e**) HRTEM image of a polycrystalline brookite seed (in green) in the second aggregate (see the rectangle on the right in panel 3**b**). The two main crystalline domains are separated by a white dashed line, and the corresponding 2D-FFTs are presented below. Both show the interplanar distances and angular relationships expected from brookite.

The first evolution of the sample was already observed at 380 °C, with seeds around 10 nm in size appearing at the nanotubes, and indicated in the low magnification TEM images (shown in Figure 3a,b) by red arrows. It should be noticed that these seeds were not observed when the same aggregates were previously imaged by low magnification TEM, as reported in Figure 2. Moreover, two-dimensional fast Fourier transform (2D-FFT) analysis of the seeds’ HRTEM images reported in Figure 3d,e confirmed that these seeds are generally polycrystalline in nature and sport lattice distances and angular relationships compatible with brookite and rutile (as shown in the lower part of panels Figure 3d,e). Moreover, a patched crystallization of the TANs was detected outside the seeds and can be observed in Figure 3c. Even if the seldom occurrence of seeds would not have been likely detected during the SAED experiment, the occurrence of zones with patched crystallization at a nominally lower temperature indicates a possible additional contribution induced by the more energetic electron beam with respect to the previous experiment. 

**Figure 4:**
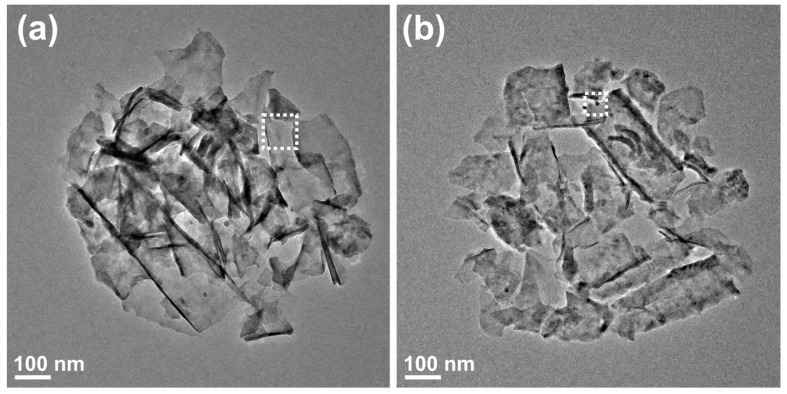

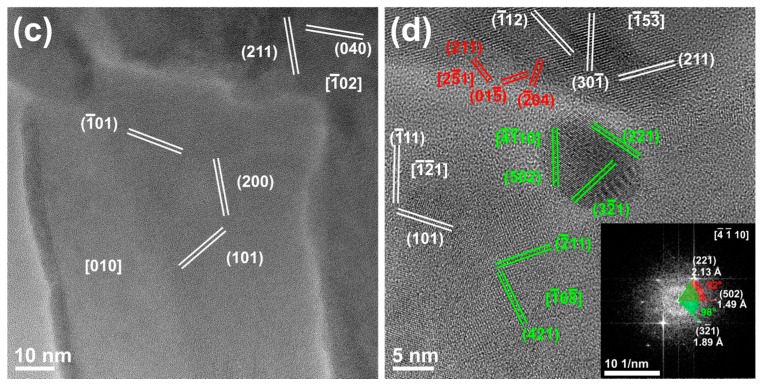
*In situ* HRTEM heating experiment of micron-sized TANs: (**a**,**b**) Evolution of the small aggregates of TANs reported in Figure 2 and Figure 3, now recorded at 500 °C. The white dotted rectangles indicate the regions of interest analyzed by HRTEM and reported in panels **c** and **d**; (**c**) HRTEM image of a an extended crystal domain of rutile (in white) in the first aggregate (see the rectangle in panel 4**a**); (**d**) HRTEM image of region of the second aggregate with patched crystallization (see the rectangle in panel 4**b**). Distinct crystal domains of rutile (in white), brookite (in green) and anatase (in red) can be identified. The 2D-FFT of the brookite seed is presented in the inset, showing its interplanar distances and corresponding angular relationships for the observed zone axis.

Figure 4 shows the TANs imaged after the temperature was further increased to 500 °C, in order to reach conditions corresponding to the final crystallization of the sample. The seeds were still present and showing a slight increase in size and roundness, while the nanotubes had evolved to polycrystals. More in detail, the seeds were still polycrystalline, albeit with bigger-sized domains of rutile and brookite phases, while the nanotubes featured a disordered crystallization, featuring extended crystal domains along with smaller, disordered, multi-domain zones; the most extended crystal domains could be identified by 2D-FFT analysis as rutile, with smaller zones and islands also showing structural features of brookite and anatase. 

**Scheme 1:**
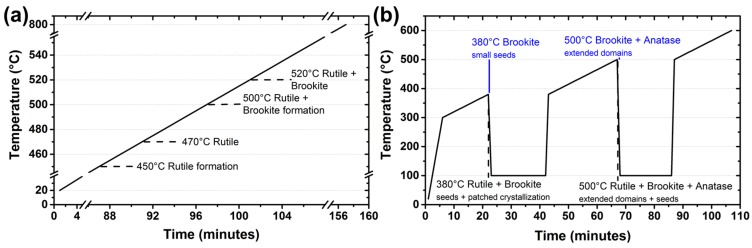
Time vs. temperature schemes depicting the thermal ramps used during the *in situ* heating studies: (**a**) thermal ramp used during the *in situ* SAED heating experiment; (**b**) thermal ramp used during the *in situ* HRTEM heating experiment. Milestones in black at the bottom of panel (**b**) indicate the structural evolution of the main population of TANs (250 nm in length, see Figure 2, Figure 3 and Figure 4); milestones in blue at the top of panel (**b**) indicate the structural evolution of the secondary population (around one micron in length, see Figure 5). The pressure value in both the experiments was less than 10^−5^ Pa.

**Figure 5:**
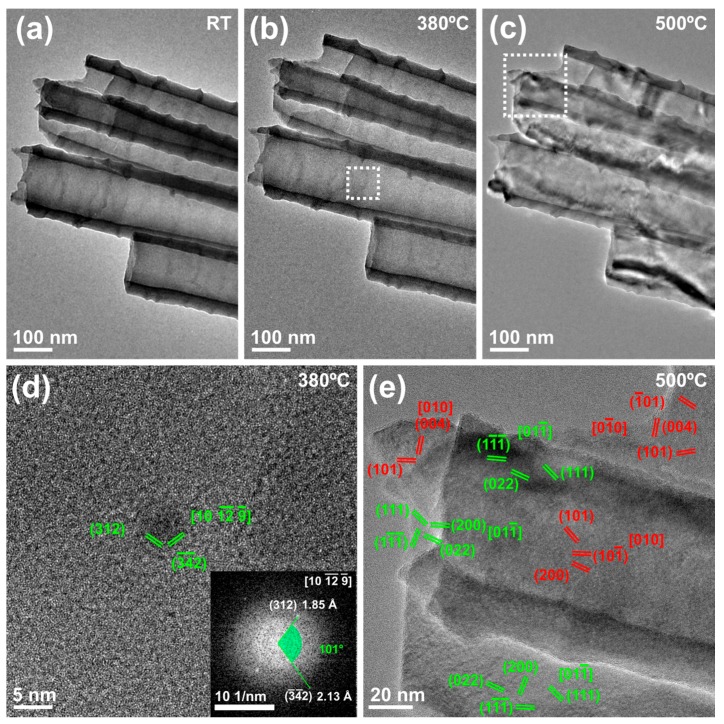
*In situ* HRTEM heating experiment of bigger TANs: (**a**–**c**) Evolution of the aggregates of TANs recorded at RT (**c**), 380 °C (**b**) and 500 °C (**c**), The white dotted rectangles indicate the regions of interest analyzed by HRTEM and reported in panels **d** and **e**; (**d**) HRTEM image of a brookite seed (in green) recorded at 380 °C (see the rectangle in panel 3**b**). The inset presents the corresponding 2D-FFT, showing the interplanar distances and angular relationships expected for the brookite; (**d**) HRTEM image of extended crystal domains of brookite (in green) and anatase (in red) recorded at 500 °C (see the rectangle in panel 4**c**).

Moreover, a group of bigger TANs, with a mean diameter of more than 200 nm and a length above one micron, showed a different structural evolution with respect to their smaller counterpart, displayed in Figure 5. These nanotubes did not present any structural evolution at 380 °C apart from showing very few 5-nm-sized seeds (Figure 5b,d), while at 500 °C they presented wider and more ordinate crystal domains of brookite and anatase (Figure 5c,e). The dissimilarity between their structural evolution and that of the nanotubes on the one hand, and of the general evolution of the aggregates on the other hand, suggest that the bigger TiO_2_ tubes represent a minor part of the sample, while their crystallization in anatase and brookite phases over rutile and brookite could be attributed to a temperature driven, size-related effect. Finally, no trace of materials coming from reduced TiO_2_ was found in any region of the examined aggregates. The thermal profiles used in both the SAED-based and HRTEM-based experiments are shown in the Scheme 1, where both the crystal phases appearance and their thermal evolution, as described above, are also summarized.

## 3. Discussion

Recently, we carried out an in-depth study of the effect of TAN crystallization on their mechanical properties [28]. In that case the TAN heating was performed *ex situ*, i.e., under ambient pressure conditions outside the electron microscope, and the TANs were subsequently imaged by HRTEM at RT after they reached a temperature of 150 , 300 and 450 °C, respectively. The structural evolution measured at these temperatures was then compared with that studied by means of both HRTEM and Raman spectroscopy. We found that the TAN crystallization had just started at 150 °C, with the appearance of very small (2–3 nm) crystalline domains of anatase. At 300 °C, their size grew to about 10 nm, while some further and quite smaller domains of brookite also appeared. Finally, at 450 °C crystallization was observed to have further proceeded, with the TANs being constituted by large zones of anatase, even if brookite was still present. As the HRTEM provided a very local structural characterization obtained by 2D-FFT analysis, the Raman spectroscopy, performed over a much larger scale, allowed the determination of the anatase as the majority phase. These results basically confirm what was already reported both in [22,23], where TANs of similar size to ours were thermally crystallized under ambient pressure conditions, as reported in the introduction. It is noteworthy that in the two cases the observed evolution is very similar in terms of starting temperature of the amorphous-to-crystalline transition, as well as the kind of crystalline phase formed and the growth observed in the size of crystalline domains. Conversely, when performed *in situ*, i.e., inside the electron microscope, the same heating experiment leads to different outcomes due to the variation in vacuum conditions (*p* < 10^−5^ Pa). First, under such a low pressure the TANs underwent a transition to rutile and brookite, with the crystallization in general starting at higher temperatures than those determined in all the previous heating experiments conducted under standard pressure. Then, even if the intrinsically very local nature of HRTEM imaging does not allow the provision of a precise quantitative determination of the majority crystal phase appearing as a consequence of the thermal treatment, some rough consideration may be attempted about the relative prominence of the crystal phases we observed. In fact, looking at the HRTEM results reported in Figure 2, Figure 3 and Figure 4, it appears quite clearly that most of the smaller TANs crystallize in polycrystalline rutile nanotubes, with the SAED-based results reported in Figure 1 confirming what was locally observed by HRTEM. Conversely, the thermal evolution of the rarer and bigger TANs reported in Figure 5 lead to the crystallization in both the anatase and brookite phases, in a similar fashion to what observed in the case of physically confined and self-confined TANs under ambient pressure conditions [23]. To our best knowledge, this is the first case in which the TANs amorphous-to-crystalline phase transition has been investigated under such a low-pressure conditions, although similar phase variation processes have already been observed at room temperature under lower vacuum conditions (*p* ≈ 10^−3^ Pa) due to visible light irradiation [29,30]. 

Furthermore, changing the electron beam energy of our TEM experiment (from 120 keV for the first, ED-based observation to 300 keV for the C_s_-corrected HRTEM imaging) gives rise to a pronounced decrease of the TANs’ initial crystallization temperature, indicating an electron beam contribution to the phenomenon. The several effects caused by the electron beam irradiation of a sample have been extensively studied, see for instance [31,32], and among them specimen heating is the one that could be most immediately considered in our case. However, if this was the only effect to take into account, the sample should undergo a local thermal increase at least equal to the difference in temperature observed when the electron beam energy (*E*_0_) changed from 120 to 300 keV (i.e., Δ*T* = 450 °C − 380 °C = 70 °C for Δ*E* = 180 keV) in order to give rise to the lower crystallization temperature observed at 300 keV. Conversely, electron beam-induced sample heating is a well-known phenomenon caused by inelastic electron–electron scattering, capable of raising the sample temperature by only a few degrees [31]. Then, it cannot provide the additional thermal contribution needed to decrease the temperature of crystallization that we observed. Moreover, even if Δ*T* is usually considered directly proportional to Δ*E*, the total amount of energy lost by the electron beam decreases with the increasing energy, due to the reduced electron–atom scattering cross-section. For the same reason, the radiolysis effects, which consist in bond breaking, decrease when *E*_0_ increases, and cannot be considered as playing a role in the lower crystallization temperature of the TANs. Conversely, such a decrease has to be ascribed to atomic displacement, also known as the knock-on effect, suffered by the sample atoms when the high-energy beam electrons hit them. In this case, the maximum amount of energy *E*_m_ transferred between fast electrons and atoms is related to *E*_0_ by the following equation [33]:

*E*_m_ = 2*E*_0_(*E*_0_ + 2*m*_0_*c*^2^)/*Mc*^2^, (1)

where *c* is the light speed, *m*_0_ the electron rest mass and *M* the target atom’s mass. Then, the knock-on effect takes place whenever the displacement energy is lower than the transferred energy, with the mass of the target atom being the only sample-related parameter and atoms with lower mass being more likely to suffer from atomic displacement. The dependence of the starting temperature of crystallization on the electron beam energy *E*_0_ was also previously observed for different materials, namely Si_2_Sb_2_Te_5_ [34] and Ge_2_Sb_2_Te_5_ [35]. In both cases, the materials amorphous-to-crystalline phase transition was promoted by an increase of *E*_0_, as a consequence of atomic displacement induced by the electron beam irradiation. It is noteworthy to highlight that the change in *E*_0_ affected just the starting temperature of crystallization, not the crystalline phases formed (brookite and rutile) nor the final polycrystalline nature of the crystallized nanotubes.

Also, our results are in good accordance with those obtained by non-thermal, optically-assisted crystallization of amorphized TiO_2_ nanocrystals to the rutile phase under oxygen-poor conditions [30]. There, an adequately energetic irradiation gave rise to oxygen desorption processes that improved the superficial chemical reactivity of TiO_2_ and triggered the crystallization of neighboring amorphous nanoparticles into bigger rutile nanocrystalline seeds. Here, the variation in the electron beam energy from 120 to 300 keV increases the displacement of oxygen atoms, thus affecting the chemical reactivity at the surface of TiO_2_ nanotubes and leading to the decrease in crystallization temperature that we observed, while also providing an indication with regards to the rare formation of the nanoseeds observed in Figure 3, Figure 4 and Figure 5. Local variations in the quantity of displaced oxygen atoms could render the amorphous TiO_2_ locally unstable and trigger the formation of small nanocrystalline precursors (<2 nm) that act as a starting point for both nanoseeds and patched crystallization zones. Then, the occurrence of nanoseeds could derive from the fast sintering of two or more nearby precursors [36,37], that coalesce to reach an energetically favorable condition and are not further modified by the heating. The appearance of rutile nanoseeds might be caused by the direct nucleation of at least one rutile precursor, which will command the crystallization during the fast sintering [38], while brookite nanoseeds should form in presence of at least one anatase precursor [36]. Then the formation of brookite seeds, albeit unexpected according to the TiO_2_ phase diagram under standard pressure conditions, suggests a local effect of the amorphous-to-crystalline transition under high vacuum conditions. These results require further experimental and theoretical studies to better comprehend how such low pressure conditions influence the phase diagram of TiO_2_ at the nanosize.

Obviously, understanding why different crystal phases appear and stabilize in the shorter or longer TANs crystallized under high vacuum, or depending on the pressure conditions adopted during heating, is not trivial. In fact, it is well known that crystallization occurs via a collective atom displacement, following the two steps of nucleation and subsequent growth. Since the phenomenon is basically driven by the crystallization enthalpy and the free volume reduction, an in-depth understanding of their dependence on the external pressure would be needed to fully comprehend the differences observed when the TANs amorphous-to-crystalline phase transition is performed under high vacuum (i.e., lower than 10^−5^ Pa). With this aim, further theoretical studies will need to be performed to elucidate more in-depth the origin of the pressure-dependent differences we observed and these will be presented in a separate paper.

## 4. Materials and Methods

A Ti foil was used as a working electrode for the anodic growth of TiO_2_ nanotube arrays in a two-electrode configuration. Anodic oxidation was carried out in an ethylene glycol electrolyte containing 0.5 wt % NH_4_F and 2.5 vol % deionized water under a constant voltage of 60 V for 0.5 h using a Direct current (DC) power supply. The samples were then rinsed in Deionized Water (DI-water), dried and detached by ultra-sonication in ethanol for five minutes. 

TiO_2_ amorphous nanotubes were studied by *in situ* TEM heating through two different sets of experiments. For both the experiments, the amorphous nanotubes were dispersed in distilled water and then drop-casted on silicon nitride based-MEMS, which also acted as the TEM heating substrate. First, an electron diffraction imaging was conducted on nanotubes aggregates using a Tecnai TEM by FEI (Hillsboro, OR, USA), equipped with a lanthanum exaboride thermionic electron source, a twin objective lens and a Orius CCD camera by Gatan (Pleasanton, CA, USA). This microscope worked at an acceleration voltage of 120 kV. In this case the *in situ* heating (5 °C·min^−1^) was performed in the RT-to-800 °C thermal range in order to assess the crystallization temperature. Second, the HRTEM imaging was performed using a spherical aberration (C_S_) corrected Titan microscope by FEI (Hillsboro, OR, USA), equipped with a Schottky field emission gun (FEG), a CEOS spherical aberration corrector of the objective lens, a 2K × 2K CCD camera by Gatan (Pleasanton, CA, USA), and with the microscope working at an acceleration voltage of 300 kV. In this case the *in situ* heating (5 °C·min^−1^) was performed in the 300-to-600 °C thermal range, after having fast reached (*t* < 300 s) the starting temperature of 300 °C. The temperature was fast lowered to 100 °C for the acquisition of the HRTEM images corresponding to each heating temperature and consequently restored before continuing the *in situ* heating. Both experiments were performed using a Wildfire MEMS-based *in situ* heating TEM sample holder by DENSsolutions (Delft, Netherlands). The pressure value in both the electron microscopes was less than 10^−5^ Pa. A structural characterization was performed to calculate the interplanar distances and identify crystal phases: in the case of the SAED patterns it was carried out by measuring the point-to-point distance of diffraction rings and diffraction spots and calculating the correspondent interplanar distances; in the case of the HRTEM images, interplanar distances and angular relationships were more precisely obtained by two-dimensional fast Fourier transform (2D-FFT) analysis of regions of interests.

## 5. Conclusions

In summary, our work showed how TAN crystallization occurs under high vacuum conditions by *in situ* heating them in a TEM. When irradiated with electrons accelerated up to the energy of 120 keV, the starting temperature of crystallization was equal to 450 °C, while if the same *in situ* heating was performed under an electron beam of 300 keV, this temperature decreased to 380 °C, due to the atomic displacement suffered by the sample atoms as a consequence of the higher electron energy, which did not affect the formation of different crystalline phases. Besides, it was also shown with unprecedented detail how TAN crystallization occurs, starting from small brookite and rutile crystalline seeds that then grow in size during the following further heating. A comparison with the same phenomenon studied under standard pressure conditions highlighted two main differences: first, the transition starting temperature was higher, and second, the crystalline phases formed were brookite and rutile rather than anatase. Furthermore, this study opens an unprecedented opportunity to study the TiO_2_ crystallization under high-vacuum conditions. This could also allow preferential access to crystalline phases that are normally forbidden when the thermally-driven crystallization takes place under ambient pressure conditions.

## References

[1] Long H., Chen A., Yang G., Li Y., Lu P. (2009). Third-order optical nonlinearities in anatase and rutile TiO_2_ thin films. Thin Solid Films.

[2] Dürr M., Schmid A., Obermaier M., Rosselli S., Yasuda A., Nelles G. (2005). Low-temperature fabrication of dye-sensitized solar cells by transfer of composite porous layers. Nat. Mater..

[3] Yang M.-Q., Xu Y.-J. (2013). Selective photoredox using graphene-based composite photocatalysts. Phys. Chem. Chem. Phys..

[4] Page K., Palgrave R.G., Parkin I.P., Wilson M., Savin S.L.P., Chadwick A.V. (2007). Titania and silver–titania composite films on glass—Potent antimicrobial coatings. J. Mater. Chem..

[5] Deng D., Kim M.G., Lee L.Y., Cho J. (2009). Green energy storage materials: Nanostructured TiO_2_ and Sn-based anodes for lithium-ion batteries. Energy Environ. Sci..

[6] Crossland E.J.W., Noel N., Sivaram V., Leijtens T., Alexander-Webber J.A., Snaith H.J. (2013). Mesoporous TiO_2_ single crystals delivering enhanced mobility and optoelectronic device performance. Nature.

[7] Guldin S., Hüttner S., Tiwana P., Orilall M.C., Ülgüt B., Stefik M., Docampo P., Kolle M., Divitini G., Ducati C. (2011). Improved conductivity in dye-sensitised solar cells through block-copolymer confined TiO_2_ crystallization. Energy Environ. Sci..

[8] Tétreault N., Horváth E., Moehl T., Brillet J., Smajda R., Bungener S., Cai N., Wang P., Zakeeruddin S.M., Forró L. (2010). High-efficiency solid-state dye-sensitized solar cells: Fast charge extraction through self-assembled 3D fibrous network of crystalline TiO_2_ nanowires. ACS Nano.

[9] Adachi M., Murata Y., Takao J., Jiu J., Sakamoto M., Wang F. (2004). Highly efficient dye-sensitized solar cells with a titania thin-film electrode composed of a network structure of single-crystal-like TiO_2_ nanowires made by the “oriented attachment” mechanism. J. Am. Chem. Soc..

[10] Jennings J.R., Ghicov A., Peter L.M., Schmuki P., Walker A.B. (2008). Dye-sensitized solar cells based on oriented TiO_2_ nanotube arrays: Transport, trapping, and transfer of electrons. J. Am. Chem. Soc..

[11] Fabregat-Santiago F., Barea E.M., Bisquert J., Mor G.K., Shankar K., Grimes C.A. (2008). High carrier density and capacitance in TiO_2_ nanotube arrays induced by electrochemical doping. J. Am. Chem. Soc..

[12] Docampo P., Guldin S., Steiner U., Snaith H.J. (2013). Charge transport limitations in self-assembled TiO_2_ photoanodes for dye-sensitized solar cells. J. Phys. Chem. Lett..

[13] Choi S.Y., Mamak M., Speakman S., Chopra N., Ozin G.A. (2004). Evolution of nanocrystallinity in periodic mesoporous anatase thin films. Small.

[14] Kondo J.N., Domen K. (2008). Crystallization of mesoporous metal oxides. Chem. Mater..

[15] Lu X., Wang G., Zhai T., Yu M., Gan J., Tong Y., Li Y. (2012). Hydrogenated TiO_2_ nanotube arrays for supercapacitors. Nano Lett..

[16] Liu Z., Zhang X., Nishimoto S., Jin M., Tryk D.A., Murakami T., Fujishima A. (2008). Highly ordered TiO_2_ nanotube arrays with controllable length for photoelectrocatalytic degradation of phenol. J. Phys. Chem. C.

[17] Gui Q., Xu Z., Zhang H., Cheng C., Zhu X., Yin M., Song Y., Lu L., Chen X., Li D. (2014). Enhanced photoelectrochemical water splitting performance of anodic TiO_2_ nanotube arrays by surface passivation. ACS Appl. Mater. Interfaces.

[18] Park J., Bauer S., Von Der Mark K., Schmuki P. (2007). Nanosize and vitality: TiO_2_ nanotube diameter directs cell fate. Nano Lett..

[19] Nicthová L., Kužel R., Matêj Z., Šícha J., Musil J. (2009). Time and thickness dependence of crystallization of amorphous magnetron deposited TiO_2_ thin films. Z. Kristallogr. Suppl..

[20] Kužel R., Nicthová L., Matêj Z., Herman D., Šícha J., Musil J. (2007). Study of crystallization of magnetron sputtered TiO_2_ thin films by X-ray scattering. Z. Kristallogr. Suppl..

[21] Kužel R., Nicthová L., Matêj Z., Herman D., Šícha J., Musil J. (2007). Growth of magnetron sputtered TiO_2_ thin films studied by X-ray scattering. Z. Kristallogr. Suppl..

[22] Oh H.-J., Lee S., Lee B., Jeong Y., Chi C.-S. (2011). Surface characteristics and phase transformation of highly ordered TiO_2_ nanotubes. Met. Mater. Int..

[23] Kim M., Bae C., Kim H., Yoo H., Montero Moreno J.M., Jung H.J., Bachmann J., Nielsch K., Shin H. (2013). Confined crystallization of anatase TiO_2_ nanotubes and their implications on transport properties. J. Mater. Chem. A.

[24] Xue C., Narushima T., Ishida Y., Tokunaga T., Yonezawa T. (2013). Double-wall TiO_2_ nanotube arrays: Enhanced photocatalytic activity and *in situ* TEM observations at high temperature. ACS Appl. Mater. Interfaces.

[25] Yuan W., Wang Y., Li H., Wu H., Zhang Z., Selloni A., Sun C. (2015). Real-time observation of reconstruction dynamics on TiO_2_ (001) surface under oxygen via an environmental transmission electron microscope. Nano Lett..

[26] Park H., Jie H.S., Kim K.H., Ahn J.P., Park J.K. (2007). *In-Situ* TEM observation on phase formation of TiO_2_ nanoparticle synthesized by flame method. Materials Sci. Forum.

[27] Ghassemi H., Harlow W., Mashtalir O., Beidaghi M., Lukatskaya M.R., Gogotsi Y., Taheri M.L. (2014). *In situ* environmental transmission electron microscopy study of oxidation of two-dimensional Ti_3_C_2_ and formation of carbon-supported TiO_2_. J. Mater. Chem. A.

[28] Stassi S., Lamberti A., Roppolo I., Casu A., Bianco S., Scaiola D., Falqui A., Pirri C.F., Ricciardi C. (2017). Evolution of nanomechanical properties and crystallinity of individual titanium dioxide nanotube resonators. Nanotechnology.

[29] Ricci P.C., Casu A., Salis M., Corpino R., Anedda A. (2010). Optically controlled phase variation of TiO_2_ nanoparticles. J. Phys. Chem. C.

[30] Ricci P.C., Carbonaro C.M., Stagi L., Salis M., Casu A., Enzo S. (2013). Delogu, anatase-to-rutile phase transition in TiO_2_ nanoparticles irradiated by visible light. J. Phys. Chem. C.

[31] Egerton R.F., Li P., Malac M. (2004). Radiation damage in the TEM and SEM. Micron.

[32] Egerton R.F. (2013). Control of radiation damage in the TEM. Ultramicroscopy.

[33] Hobbs L.W., Hren J.J., Goldstein J.I., Joy D.C. (1979). Radiation effects in analysis of inorganic specimen by TEM. Introduction to Analytical Electron Microscropy.

[34] Zhang T., Song Z., Sun M., Liu B., Feng S., Chen B. (2008). Investigation of electron beam induced phase change in Si_2_Sb_2_Te_5_ material. Appl. Phys. A.

[35] Kooi B.J., Groot W.M.G., De Hosson J.T.M. (2004). *In situ* transmission electron microscopy study of the crystallization of Ge_2_Sb_2_Te_5_. J. Appl. Phys..

[36] Mao Q., Ren Y., Luo K.H., Li S. (2015). Sintering-induced phase transformation of nanoparticles: A molecular dynamics study. J. Phys. Chem. C.

[37] Zhou Y., Fichthorn K.A. (2012). Microscopic view of nucleation in the anatase-to-rutile transformation. J. Phys. Chem. C.

[38] Koparde V.N., Cummings P.T. (2008). Phase Transformations during sintering of titania nanoparticles. ACS Nano.

